# Editorial: Global excellence in neural circuits: Central and South America

**DOI:** 10.3389/fncir.2024.1432862

**Published:** 2024-07-08

**Authors:** Cristina Martins-Silva

**Affiliations:** Health Sciences Center, Federal University of Espírito Santo, Vitoria, Espirito Santo, Brazil

**Keywords:** neural circuits, neuronal network, corticofugal pathway, prefrontal cortex (PFC), behavioral flexibility

The nervous system comprises two cell types: neurons and glia. Individually or collectively, these cells form interconnected systems with different levels of complexity—neural circuits—that build the perception of the external world and guide the decisions made by each species. From this simplistic perspective, one may ask: how do these cells contribute to different behaviors and how does the environment influence them? To address these questions, we can begin with the notion proposed by Hippocrates that in order to properly study the mind, one must first study the brain.

The organization of the brain shows how this group of cells forms an intricate and well-orchestrated circuitry. The neuropil (or neuropile) is a fundamental form of cellular organization in the brain, in which a dense network of dendrites, axonal terminals, and glial processes connect in a precise and finely coordinated manner. Therefore, comprehending how information flows in a specific circuit is essential for understanding its function.

The in-depth understanding of neural circuits began with cellular studies conducted by Camillo Golgi and Santiago Ramon Y. Cajal, progressing to the breakthrough in neurophysiology with the publication of the book “Integrative action of the nervous system” by Sir Charles Scott Sherrington that coined the term synapse. Currently, gene manipulations in specific cell groups, coupled to optogenetics tools, enable the evaluation of how cells within a specific circuitry contribute to precise behaviors. For instance, studies examine the persistent chemical and or/physical changes that occur in the nervous system—engrams—in response to learned experiences and how these engrams are involved in the encoding, consolidation, and retrieval of a particular memory. Therefore, modern neuroscience aims to understand how different neural circuits are interconnected and integrate within the brain, employing comprehensive maps known as connectomics.

The aim of the present editorial is to present two studies on different neural circuities in humans and animals conducted in South America. Manuscripts include one original research study and one review.

Donoso-San Martín et al. evaluated possible alterations in the corticofugal pathways in patients with tinnitus during a cross-modal attention task. The authors presented evidence that chronic tinnitus sufferers exhibit a high prevalence of neuropsychiatric conditions along with attention impairment. To test their hypothesis, the authors submitted 28 participants (14 subjects with tinnitus and 14 subjects comprising the control group) to an auditory and visual selective task using simultaneous electroencephalogram (EEG) and distortion-product otoacoustic emissions (DPOAE) recording. They proposed that this attentional paradigm, along with the aforementioned integrated recording methods, may detect possible alterations in the cerebral cortex to cochlea neural network. Although the study is limited by the small number of subjects, the authors found a significant decrease in the DPOAE oscillatory amplitude during the visual attention period, compared to the auditory period in both tinnitus and control groups. Although between-subject statistical analysis (tinnitus vs. control) was not significant, the study suggested that, in tinnitus patients, the top-down infrasonic low-frequency cochlear oscillatory activity in the delta and theta bands is preserved.

Negrón-Oyarzo et al. put forward a well-documented review on the importance of prefrontal cortex (PFC) activity, along with other important brain structures, in influencing cognitive control of behavior in normal aging and pathological mental conditions. The authors presented a large body of evidence indicating that cognitive control of behavior is directed toward goal achievement and requires the coordination of several simultaneous neural processes affected by the environment. As environmental conditions constantly change, this continuous inconstance enables animals to respond in an “elastic way”, that is, with behavioral flexibility.

Initially, to address the importance of the neural network under PFC coordination, the authors compiled several studies in rodents showing that the medial-PFC (mPFC) is a “functional homolog” to the human PFC, despite not sharing anatomical features with the primate PFC. In fact, based on cortical folding, rodents are lissencephalic and primates are gyrencephalic species. However, transcriptomic studies showed that rodents and primates exhibit similarities in laminar-gene expression, cell density, and local and large-scale connectivity. Moreover, rodents and primates are social animals, and from a behavioral perspective both species required several cognitive strategies supported by the PFC for goal-directed adaptation.

In the second part of the review article, the authors presented the neurophysiological basis of how the PFC contributes to the implementation of cognitive control of behavior. Relying on the neuronal assembly hypothesis, they argue that a small set of neurons fire synchronously during a behavioral task (e.g., the hippocampal place cells that fire specifically when the animal is at a certain location) and the synchronized neuronal spiking in the mPFC (oscillatory frequencies) seems to support cognitive operations. The amplitude and frequency of oscillations depend on the identity and neural composition of neurons in the cerebral space. Thus, different oscillatory frequencies reflect the synchronized recruitment of different levels of neural populations: large-scale neuronal populations exhibit low-frequency oscillations and local neural populations present high-frequency oscillations. As an example, the authors remarked that the mPFC exhibit theta oscillations (6–12 Hz) as the most prominent low-frequency oscillatory activity during locomotion and high cognitive demands.

Finally, in the third part of the review, the authors explore how impairments of the mPFC lead to dysfunctional activity in normal aging and mental illnesses such as mood disorders and schizophrenia. The aging of populations is rapidly accelerating worldwide. Aging is a natural process accompanied by a decline in cognitive abilities, and although it differs from pathological brain disorders, both processes share pivotal alterations in the mPFC. The authors present studies conducted in rodent models of aging and mental illness that support a robust relationship between aberrant oscillatory activity in the mPFC and the impaired cognitive control of behavior observed in elderly people and mental illness patients. The current pharmacotherapy for cognitive decline (in both normal and pathological mental conditions) is debatable, prompting the authors to propose that new studies should focus on the re-establishment of prefrontal neural activity patterns, and that rodents are suitable for pre-clinical trials.

In summary, having a collection of works about neural circuits developed by researchers from South America comprises and important source of information to inspire neuroscientists abroad.

## Author contributions

CM-S: Writing – original draft.

